# Comparison of the Real-World Reporting of Symptoms and Well-Being for the HER2-Directed Trastuzumab Biosimilar Ogivri With Registry Data for Herceptin in the Treatment of Breast Cancer: Prospective Observational Study (OGIPRO) of Electronic Patient-Reported Outcomes

**DOI:** 10.2196/54178

**Published:** 2024-04-04

**Authors:** Andreas Trojan, Sven Roth, Ziad Atassi, Michael Kiessling, Reinhard Zenhaeusern, Yannick Kadvany, Johannes Schumacher, Gerd A Kullak-Ublick, Matti Aapro, Alexandru Eniu

**Affiliations:** 1 Department of Clinical Pharmacology and Toxicology University Hospital Zurich University of Zurich Zurich Switzerland; 2 BrustZentrum Zürichsee Horgen Switzerland; 3 Faculty of Medicine University of Zurich Zurich Switzerland; 4 Onkologie Spital Oberwallis Brig Switzerland; 5 Mobile Health AG Zurich Switzerland; 6 Palleos Healthcare Wiesbaden Germany; 7 Cancer Center Clinique de Genolier Genolier Switzerland; 8 Hôpital Riviera-Chablais Rennaz Switzerland

**Keywords:** breast cancer, biosimilar, trastuzumab, electronic patient-reported outcome, ePRO, medidux, app

## Abstract

**Background:**

Trastuzumab has had a major impact on the treatment of human epidermal growth factor receptor 2 (HER2)-positive breast cancer (BC). Anti-HER2 biosimilars such as Ogivri have demonstrated safety and clinical equivalence to trastuzumab (using Herceptin as the reference product) in clinical trials. To our knowledge, there has been no real-world report of the side effects and quality of life (QoL) in patients treated with biosimilars using electronic patient-reported outcomes (ePROs).

**Objective:**

The primary objective of this prospective observational study (OGIPRO study) was to compare the ePRO data related to treatment side effects collected with the medidux app in patients with HER2-positive BC treated with the trastuzumab biosimilar Ogivri (prospective cohort) to those obtained from historical cohorts treated with Herceptin alone or combined with pertuzumab and/or chemotherapy (ClinicalTrials.gov NCT02004496 and NCT03578731).

**Methods:**

Patients were treated with Ogivri alone or combined with pertuzumab and/or chemotherapy and hormone therapy in (neo)adjuvant and palliative settings. Patients used the medidux app to dynamically record symptoms (according to the Common Terminology Criteria for Adverse Events [CTCAE]), well-being (according to the Eastern Cooperative Oncology Group Performance Status scale), QoL (using the EQ-5D-5L questionnaire), cognitive capabilities, and vital parameters over 6 weeks. The primary endpoint was the mean CTCAE score. Key secondary endpoints included the mean well-being score. Data of this prospective cohort were compared with those of the historical cohorts (n=38 patients; median age 51, range 31-78 years).

**Results:**

Overall, 53 female patients with a median age of 54 years (range 31-87 years) were enrolled in the OGIPRO study. The mean CTCAE score was analyzed in 50 patients with available data on symptoms, while the mean well-being score was evaluated in 52 patients with available data. The most common symptoms reported in both cohorts included fatigue, taste disorder, nausea, diarrhea, dry mucosa, joint discomfort, tingling, sleep disorder, headache, and appetite loss. Most patients experienced minimal (grade 0) or mild (grade 1) toxicities in both cohorts. The mean CTCAE score was comparable between the prospective and historical cohorts (29.0 and 30.3, respectively; mean difference –1.27, 95% CI –7.24 to 4.70; *P*=.68). Similarly, no significant difference was found for the mean well-being score between the groups treated with the trastuzumab biosimilar Ogivri and Herceptin (74.3 and 69.8, respectively; mean difference 4.45, 95% CI –3.53 to 12.44; *P*=.28).

**Conclusions:**

Treatment of patients with HER2-positive BC with the trastuzumab biosimilar Ogivri resulted in equivalent symptoms, adverse events, and well-being as found for patients treated with Herceptin as determined by ePRO data. Hence, integration of an ePRO system into research and clinical practice can provide reliable information when investigating the real-world tolerability and outcomes of similar therapeutic compounds.

**Trial Registration:**

ClinicalTrials.gov NCT05234021; https://clinicaltrials.gov/study/NCT05234021

## Introduction

Biosimilars and reference biologics play a key role in the treatment of cancer and account for approximately 70% of the growth in costs of drugs from 2010 to 2015 [1]. Therefore, pricing is an important challenge for the medical society and biosimilars offer an attractive option for a value-based care environment with cost-saving potential [2].

Trastuzumab (Herceptin), a human epidermal growth factor receptor 2 (HER2) antibody, has had a major impact on the treatment of patients with HER2-positive breast cancer (BC) worldwide, which now has indications for the treatment of small tumors in both (neo)adjuvant and palliative settings [3,4]. This provides a good opportunity to compare the efficacy and safety of trastuzumab biosimilars to those of trastuzumab in clinical trials. For several anti-HER2 biosimilars, safety and clinical equivalence to the reference product have been demonstrated [2,5]. In a randomized, parallel-group phase 3 equivalence study of patients with HER2-positive metastatic BC, Rugo et al [6] demonstrated equivalent efficacy and similar safety profiles between the trastuzumab biosimilar Ogivri (MYL-1401O) and trastuzumab (Herceptin) [6].

The enhanced assessment of electronic patient-reported outcomes (ePROs) in clinical routine and cancer trials is of growing interest [7-9]. Several studies indicate that the proactive use of PROs can identify otherwise undetected symptoms and improve symptom management for patients with various types of cancer [9] as well as offer improvements in well-being and awareness of adverse events (AEs) between outpatient visits. Using a mobile app, especially in collaboration with the treating physician, might improve clinical care in patients with early or advanced disease [10-13]. In addition, the benefits of digital patient monitoring have been demonstrated during immune and targeted cancer therapies in terms of more efficient symptom assessment and patient-physician communication as well as a reduced need for telephone consultations [14].

Medidux is an interactive patient empowerment app that enables physicians, especially oncologists, to monitor the progression of well-being and symptoms of patients undergoing cancer treatment. Based on the documented symptom progression, the software notifies patients to contact the treatment team if symptoms defined according to the Common Terminology Criteria for Adverse Events (CTCAE) standards are outside the acceptable range. More than 110 available symptoms and severity classifications (according to the CTCAE), as well as high numbers of standardized symptom reports from patients, contribute to the collection of high-quality ePRO data for the timely management of treatment-related AEs and toxicities and their communication to treatment teams [11,13]. Thus, the medidux smartphone app is helpful to stabilize daily functional activities and leads to more frequent reporting of AEs and more precise entries regarding symptoms [11]. The continuous measurement of ePROs enables structured and standardized data recording of patients’ daily health state.

An increased level of concordance (κ=0.68) for common symptoms, including pain, fever, diarrhea, constipation, nausea, vomiting, and stomatitis, between the patient and treating physician was recently demonstrated for the medidux platform [13].

However, to the best of our knowledge, no real-world observation of side effects, tolerability, and quality of life (QoL) has been performed using ePRO data collected from patients treated with anti-HER2 biosimilars. Thus, the aim of this observational study was to investigate real-word data on daily functional activity, symptoms, and therapy side effects recorded with the medidux smartphone app in patients undergoing Ogivri antibody therapy. In addition, historical ePRO data of 38 patients with HER2-positive BC treated with Herceptin from two previous studies [7,13] were used for comparative analysis.

## Methods

### Study Design

OGIPRO was a noninterventional, multicenter, prospective, observational study conducted at 5 study sites in Switzerland over a duration of 20 months.

Patients 18 years and older with a histologically or cytologically proven diagnosis of HER2-positive primary, locally advanced, or metastatic BC were eligible to participate after providing written informed consent. In addition, patients had to own a personal iOS or Android smartphone.

Eligible patients received anti-HER2 treatment containing the trastuzumab biosimilar Ogivri (initial dose of 8 mg/kg body weight [BW] intravenously, followed by 6 mg/kg BW) with or without pertuzumab and/or chemotherapy and hormone therapy in (neo)adjuvant and palliative settings. At the beginning of the study, patients were provided with the medidux app and were prompted to record their symptoms, well-being, EQ-5D-5L questionnaires, cognitive capabilities, and vital parameters every day. Patients underwent 3 regular study visits scheduled on days 1, 21, and 42 during their 3 weekly chemotherapeutic interventions. All anticancer treatments used in this study were approved drugs, and the therapy was compliant with national treatment guidelines.

The observational period for each patient was 6 weeks. At the end of the observational period, patients decided whether to continue their therapy with the biosimilar Ogivri or with the reference substance Herceptin.

After the study observational period, prospectively collected data of patients treated with Ogivri (prospective cohort) were compared to historical ePRO data of patients treated with Herceptin (historical cohort) in two previous studies: a prospective randomized controlled trial (PRO1 study; ClinicalTrials.gov NCT02004496) of 139 patients with early stage BC who underwent chemotherapy [7] and an observational noninterventional trial (Consilium1 study; ClinicalTrials.gov NCT03578731) of patients with breast, colon, prostate, or lung cancer undergoing cancer treatment [13]. In both studies, patients were encouraged to document data on well-being and standardized symptoms using earlier versions of the medidux app during the course of their therapies. More than 5000 continuously measured data entries from 38 patients overall (14 from Consilium1 and 24 from PRO1) were available for the comparative analysis [7,13]. The historical ePRO data were recorded in the same manner using the earlier versions of the mobile app [11] and were therefore comparable to the prospective ePRO data.

### Ethical Considerations

This study was approved by the Swiss Institutional Review Board (KEK-ZH: 2021-D0051) and was conducted in accordance with the principles of the Declaration of Helsinki (current version). The study was also registered on ClinicalTrials.gov (NCT05234021). All patients in the prospective and historical cohorts provided written informed consent prior to enrollment and were informed that participation in the study is voluntary and can be revoked at any time. All study documents were deidentified by assigning a unique ID to each patient. Functional data security was ensured by identification only made possible via the patient’s ID. The data on the patients’ devices were encapsulated in the app and the data exchange was encrypted with the patient’s ID. There was no compensation provided to participants.

### Objectives

#### Primary Objective

The primary objective of the study was to evaluate ePRO data reported in the medidux app by patients with HER2-positive BC treated with the trastuzumab biosimilar Ogivri with respect to their treatment side effects and to compare these data with ePRO data obtained from a historical cohort of 38 patients treated with Herceptin in two previous studies (NCT02004496, NCT03578731) [7,11,13]. No difference was expected for the CTCAE score between the two cohorts. The aims of the study were therefore to confirm that the average CTCAE scores were similar in both cohorts and that the recording of side effects with the app was reliable.

#### Secondary Objectives

Secondary objectives included well-being in both cohorts as well as electronically reported symptoms with respect to the therapy regimen and demographic characteristics only in the prospective cohort.

### Mobile App

The medidux app (version 3.2) used in the study is a patient-centered, therapy-accompanying app that supports the structured, standardized, and dynamic documentation of symptoms and therapy side effects. Use of this tool does not represent an invasive intervention on the patient and consequently did not pose any specific risks of physical injury.

### Data Collection

The app has two basic components: (1) a browser-based app for the treatment team (web app) and (2) a mobile app for cancer patients. There was no need for 24-hour monitoring by medical staff in connection with use of the app.

The medidux app for patients enabled recording symptoms, vital signs, and well-being in a structured and standardized manner. Patients were encouraged to regularly enter data on symptoms according to the CTCAE (version 4.0), general well-being according to the Eastern Cooperative Oncology Group Performance Status (ECOG PS), EQ-5D-5L questionnaire (weekly), vital signs (weight, blood glucose, blood pressure, and pulse), and optionally a neuropsychological cognitive test (Trail Making Test [TMT]), concomitant medications, and private notes. Patients were asked daily about their general well-being and symptoms using a visual analog scale. Recording usually started on the day of therapy initiation (or the change in therapy) and continued through an observational period of 6 weeks. The frequency of app use and data entry was logged throughout the course of the study treatment, which served as an indicator of patients’ active participation in the study and as a relevant process parameter for evaluating the usability of the app itself.

The mobile app also recommended contacting the investigator or treatment site in case of high intensity of symptoms (ie, treatment side effects). Furthermore, the app provided patients with self-efficacious recommendations and tips on how to treat and reduce treatment side effects.

### Recording of AEs

AEs in the app were classified according to the CTCAE (version 4.0). For the app, grade 5 “Death related to AE” had been removed. Instead, category 0 was added, representing no or very mild symptoms. The 5 severity levels were translated into a visual analog scale from 0.1 to 10, with 0.1 representing the weakest possible symptom and 10 representing the strongest possible symptom. Scores 0.1-2.0 corresponded to grade 0, scores 2.1-4.0 corresponded to grade 1, scores 4.1-6 corresponded to grade 2, scores 6.1-8 corresponded to grade 3, and scores 8.1-10 corresponded to grade 4 AEs. When patients selected a score between 0.1 and 10, they received a summary and information for the selected range, which was displayed in the app. Classification into adapted grades based on the CTCAE resulted in the following categories: minimal symptoms (0), mild symptoms (1), moderate symptoms (2), severe symptoms (3), and very severe symptoms (4).

### Well-Being Assessment

Self-assessment of well-being was carried out in the medidux app with the help of a slider on a visual analog scale that allows for the continuous selection from 0 to 100. At the same time, short definitions appear for the standardized and structured reporting of the gradings, which should help the patient to correctly categorize their well-being. Selected values between 81 and 100 correspond to an ECOG PS of 0, values of 61-80 correspond to ECOG PS 1, values of 41-60 correspond to ECOG PS 2, values of 21-40 correspond to ECOG PS 3, and values of 0-20 correspond to ECOG PS 4. As mentioned above, grade 5 “Dead” was removed for the app.

### Statistical Analyses

#### Sample Size Calculation

The research objective was to investigate the difference between prospective and historical cohorts regarding patient-reported side effects, operationalized by the CTCAE score over a period of 6 weeks. To assess the hypothesis of equal CTCAE scores in both cohorts, the method of interval estimation was selected using the 95% CI for the mean difference between cohorts. A statistical analysis plan (SAP) prospectively determined the required sample size for a prospective cohort based on the data from the historical cohort (as available from the previous studies NCT02004496 and NCT03578731 [7,11,13]; see the Study Design section above for further details). First, the SD for the CTCAE scores of the 38 patients in the historical cohort was calculated retrospectively as 9.7 and the assumption of an equal SD in the prospective cohort was made. Second, the sample size for the prospective cohort was chosen to achieve a certain minimum precision in estimating the mean difference between cohorts (width of the 95% CI). For a range of feasible sample sizes, the SAP reported 95% CI precisions based on the *t* distribution (calculated using the R package presize [15]), assuming an equal SD of 9.7 in both cohorts and using a pooled variance estimate. From this range, a sample size of 60 patients was prospectively selected in the SAP to achieve 51 evaluable patients, given an expected dropout rate of 15%. The corresponding 95% CI for the mean difference between the historical and prospective cohorts was estimated to have a precision of 8.3, which was deemed acceptable for the planned assessment in the given study context.

#### Statistical Methods

All analyses of the primary and secondary endpoints (CTCAE score, well-being score) were performed using univariate analyses, followed by multivariate linear regression to report (adjusted) mean differences between historical and prospective cohorts, with the *P* values based on *t* tests and corresponding 95% CIs. All multivariate models extended the respective univariate models in a supplementary fashion to adjust for potential imbalances in patient age, tumor stage, and therapy setting. These covariates were prospectively defined in the study’s SAP; no model selection procedures were employed. All analyses were performed using R version 4.2.0 (The R Foundation for Statistical Computing) [16]. Two-sided *P* values ≤.05 were considered statistically significant.

#### Primary Endpoint

The primary endpoint, a CTCAE score based on the severity grades of the 10 most relevant side effects (sensory disturbance, diarrhea, fatigue, nausea, vomiting, headache, fever, edema of the limbs, joint pain, and loss of appetite) after 6 weeks, was compared between the prospective and historical cohorts. The CTCAE score was calculated by averaging the score per patient and symptom and then averaging the score per patient over all symptoms. The mean difference in the CTCAE scores between cohorts was estimated using univariate linear regression with 95% CIs. To adjust for potential differences between the two cohorts in covariates relevant for the primary outcome, a supplementary multivariate analysis was performed including the additional covariates patient age, tumor stage, and therapy setting.

#### Secondary Endpoint

The well-being score according to the ECOG PS was collected continuously using a visual analog scale (range 0-100) implemented in the medidux app and averaged across measurements during the observational period. The analysis protocol was analogous to that described above for the primary endpoint.

#### Additional Analysis

Cognitive tests in the prospective cohort were collected continuously throughout the observation period and descriptively assessed by administering a modified version of the TMT. The time (in seconds) to complete each task (execution time) was used in the analysis.

## Results

### Baseline Characteristics

Overall, 53 female patients were enrolled in the OGIPRO study. The median age was 57 (range 34-87) years in the prospective cohort and 51 (range 31-78) years in the historical cohort. Most patients (38.9%) had tumor stage 2 (Table 1). With regard to the treatment setting, relatively fewer patients (22.2%) received palliative treatment than neoadjuvant or adjuvant treatment. More than half of the patients (59.3%) received dual anti-HER2 blockade with trastuzumab and pertuzumab (Table 1).

**Table 1:** Baseline characteristics.

Parameter		Historical cohort (n=38)		Prospective cohort (n=53)	Total (N=91)	*P* value
**Age (years)**						.001^a^
	Mean (SD)	51.3 (10)^b^	59.09 (12.193)		55.89 (11.924)^b^	
	Median (range)	51 (31-78)	57 (34-87)		54 (31-87)	
**Tumor stage (T), n (%)**						.07^c^
	T1	8 (21.05)	10 (19.23)^b^		18 (20.0)^b^	
	T2	19 (50.0)	16 (30.77)^b^		35 (38.89)^b^	
	T3	8 (21.05)	11 (21.15)^b^		19 (21.11)^b^	
	T4	3 (7.89)	15 (28.85)^b^		18 (20.0)^b^	
**Treatment setting, n (%)**						.02^c^
	Neoadjuvant	17 (44.74)	18 (34.62)^b^		35 (38.89)^b^	
	Adjuvant	18 (47.37)	17 (32.69)^b^		35 (38.89)^b^	
	Palliative	3 (7.89)	17 (32.69)^b^		20 (22.22)^b^	
**Treatment, n (%)**						.01^c^
	Trastuzumab	1 (2.63)	10 (18.87)		11 (12.09)	
	Trastuzumab+pertuzumab	21 (55.26)	33 (62.26)		54 (59.34)	
	Trastuzumab+pertuzumab+paclitaxel	13 (34.21)	10 (18.87)		23 (25.27)	
	Ado-trastuzumab emtansine	3 (7.89)	0 (0)		3 (3.3)	
**ECOG PS^d^, n (%)**						.26^c^
	0	13 (34.21)	16 (30.77)^b^		29 (32.22)^b^	
	1	15 (39.47)	29 (55.77)^b^		44 (48.89)^b^	
	2	6 (15.79)	4 (7.69)^b^		10 (11.11)^b^	
	3	2 (5.26)	3 (5.77)^b^		5 (5.56)^b^	
	4	2 (5.26)	0 (0)^b^		2 (2.22)^b^	

^a^Student *t* test.

^b^Data missing for 1 participant.

^c^*χ*^2^ test.

^d^ECOG PS: Eastern Cooperative Oncology Group Performance Status.

### ePRO Data

**Figure 1:**
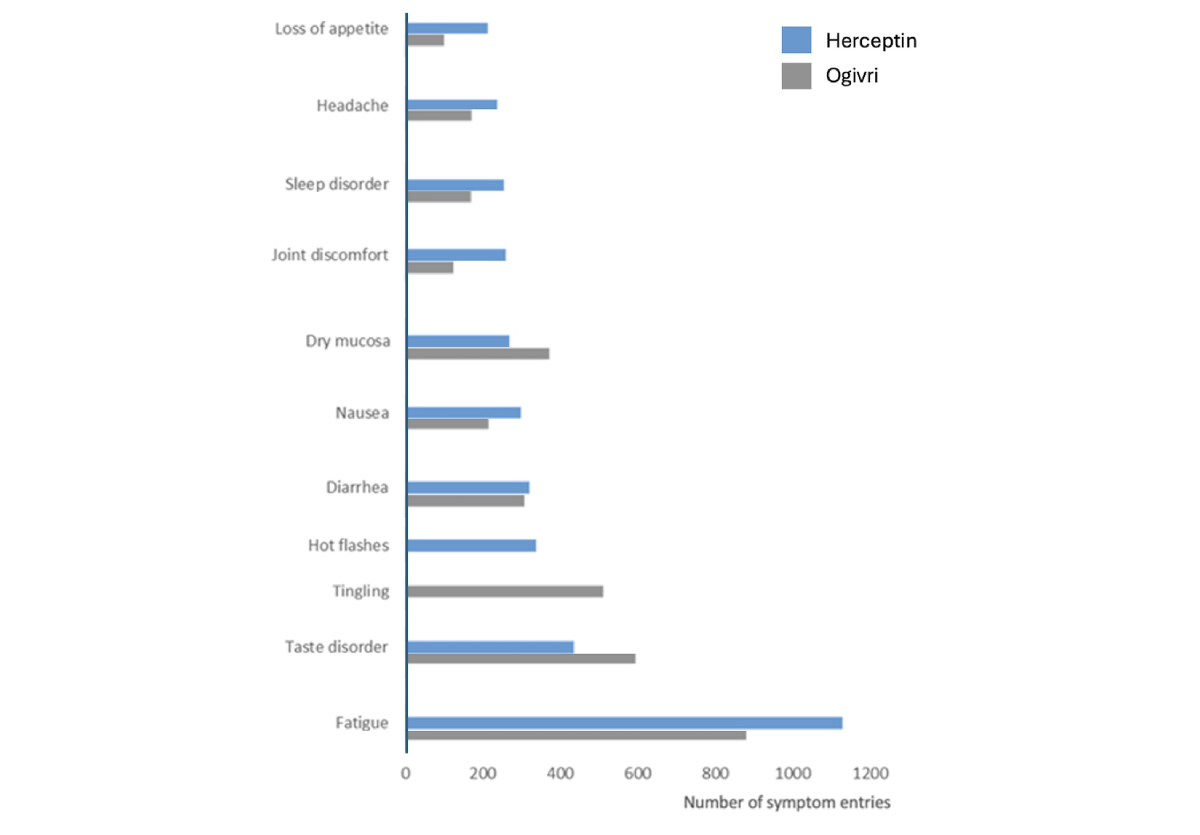
Frequency of the 10 most common symptoms recorded with the app by patients treated with the trastuzumab biosimilar Ogivri (prospective cohort) and patients treated with Herceptin (historical cohort). Absolute numbers of symptom entries are presented.

In the prospective cohort, 84 of the 92 available different symptoms were entered (average >4 symptoms/day), resulting in a total of 9680 symptoms, whereas 54 of the 82 different symptoms were reported in the historical cohort (average >3 symptoms/day), resulting in a total of 6904 symptom entries. The most common symptoms reported in both groups included fatigue, taste disorder, nausea, diarrhea, dry mucosa, joint discomfort, tingling, sleep disorder, headache, and appetite loss (Figure 1).

Overall, the distribution of symptom grades in the Ogivri cohort revealed that most patients experienced minimal (grade 0) and mild (grade 1) toxicities, followed by grade 2, grade 3, and grade 4 toxicities (Table 2). The results for QoL (based on the EQ-5D-5L questionnaire), which was also assessed in this study, will be reported elsewhere.

**Table 2:** Distribution of symptom grades in the Ogivri prospective cohort (N=9680 symptoms reported).

App symptom score and grade	Entries, n (%)
≤2 (Grade 0=minimal)	4167 (43.1)
>2 to ≤4 (Grade 1=mild)	4040 (41.7)
>4 to ≤6 (Grade 2=moderate)	1268 (13.1)
>6 to ≤8 (Grade 3=severe)	164 (1.7)
>8 to ≤10 (Grade 4=very severe)	41 (0.4)

### CTCAE Score

**Figure 2:**
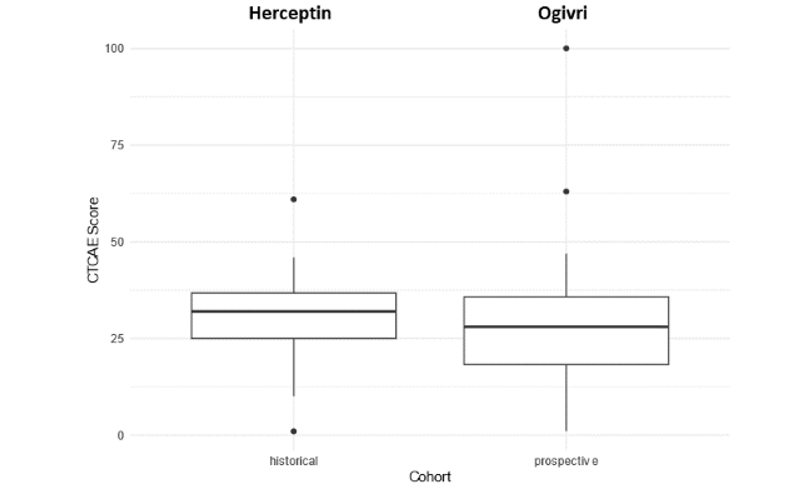
CTCAE score in the prospective (Ogivri) and historical (Herceptin) cohorts. The CTCAE score (primary endpoint) was analyzed in 50 patients (3 patients were excluded due to missing data on symptoms) treated with Ogivri and in all 38 patients treated with Herceptin. CTCAE: Common Terminology Criteria for Adverse Events.

The primary endpoint was analyzed in 50 patients (3 patients were excluded due to missing data on symptoms) treated with Ogivri (prospective cohort) and in all 38 patients treated with Herceptin (historical cohort). The mean CTCAE scores were comparable between the two cohorts (Table 3) with a mean difference of –1.27 (95% CI –7.24 to 4.70; *P*=.68) (Figure 2). In the multivariate analysis, the adjusted mean CTCAE scores also did not differ between the two cohorts (2.51, 95% CI –3.27 to 8.29) (Table S1 in Multimedia Appendix 1).

**Table 3:** Descriptive statistics of treatment side effects according to Common Terminology Criteria for Adverse Events (CTCAE) scores and well-being according to the Eastern Cooperative Oncology Group Performance Status (ECOG PS).

Parameter		Historical cohort (n=38)	Prospective cohort (n=53)	Total (N=91)	*P* value^a^
**CTCAE score^b^**					.68
	Mean (SD)	30.29 (11.618)	29.02 (15.804)	29.57 (14.088)	
	Median (range)	32 (1-61)	28 (1-100)	29.5 (1-100)	
**ECOG PS^c^**					.28
	Mean (SD)	69.82 (23.006)	74.27 (15.66)	72.39 (19.117)	
	Median (range)	76 (0-100)	74.5 (35-100)	76 (0-100)	

^a^Reported *P* values correspond to mean differences between cohorts.

^b^Missing scores for 3 participants in the prospective cohort.

^c^Missing score for 1 participant in the prospective cohort.

### Well-Being Score

The secondary endpoint, the well-being score, was analyzed in 52 patients (one patient was excluded due to missing data on well-being) from the OGIPRO study and in all 38 patients from the historical cohort. The mean well-being score did not differ significantly between patients treated with Ogivri and those treated with Herceptin (Table 3), with a mean difference of 4.45 (95% CI –3.53 to 12.44; *P*=.28). The adjusted mean well-being scores also did not differ between the two cohorts (3.78, 95% CI –4.64 to 12.19) (Table S2 in Multimedia Appendix 1).

### Cognitive Abilities in the Prospective Cohort

A total of 767 cognitive tests were entered and the data of 37 patients (70%) who had performed at least one test were included in the analysis (see Figure S1 in Multimedia Appendix 1). Overall, the mean execution time was 42.9 (SD 26.3) with an absolute difference between the maximum and minimum execution time of 197 seconds. Because of the low sample size and limited number of cognitive tests recorded, no correlation analysis between cognitive abilities and treatment was performed.

## Discussion

The treatment of patients with HER2-positive BC with the trastuzumab biosimilar Ogivri resulted in equivalent symptoms, AEs, and well-being to those experienced under treatment with Herceptin as determined by ePROs. Ogivri treatment in the context of HER2-positive BC was well tolerated and no new important safety risks were observed. The results of this study are consistent with previously reported evidence on the safety comparability of the trastuzumab biosimilar Ogivri to the reference product Herceptin for the treatment of HER2-positive BC [6,17].

The use of biosimilars in oncology could reduce health care costs and thus expand access to drugs worldwide. The European Medicines Agency as well as the US Food and Drug Administration have developed guidelines requiring biosimilars to demonstrate comparable results in relevant clinical trials to those obtained using the original product [18]. Recent studies have demonstrated that anti-HER2 therapy can be switched safely to trastuzumab biosimilars and successfully implemented in clinical practice [19].

In our study, the incidence and distribution of symptoms associated with Ogivri were similar to those reported with Herceptin. However, the slightly lower mean symptom score related to Ogivri might be attributed to the higher number of treatments in this cohort for advanced cancer stages, including antihormone treatments and dual HER2 blockade.

To our knowledge, this study represents the first real-world evaluation on efficacy and safety in patients treated with HER2 biosimilars using ePRO data. Use of the app in this study was intended to help patients gain a better overview of their disease history and improve their symptom management. Our analysis of ePRO data demonstrated comparable CTCAE scores between the prospective Ogivri cohort and the historical Herceptin cohort. These findings further support the previously reported similar safety profiles between the trastuzumab biosimilar and the corresponding reference product [6,17] with no new safety concerns observed.

Importantly, the well-being score based on the ECOG PS did not differ between the two cohorts. In a pooled analysis of data from three randomized clinical trials including patients with HER2-positive advanced BC, PROs were identified as an independent prognostic factor for both survival and toxicity outcomes. In addition, patient-reported physical well-being and clinically interpreted ECOG PS provided independent prognostic information [20]. In our prospective Ogivri cohort, we did not focus on the prognostic value of the ePRO with regard to clinical outcomes, but we were able to demonstrate that an eHealth patient empowerment app can provide reliable information on side effects and well-being when comparing a biosimilar with reference treatments. Hence, the use of continuous eHealth-based symptom reporting together with biosimilars can result in a potential economic benefit by reducing the cost of drug treatment and hospitalization. Further detailed analyses of randomized trials with biosimilars will help to quantify these resources more comprehensively.

In general, the diary characteristic of apps might appear helpful to capture and recall disease-related information such as cognitive impairments [21]. In the OGIPRO study, patients had the possibility to complete a TMT, which is one of the most widely used neuropsychological tests in clinical practice; this test is perceptive, easy to understand for patients, has a short administration time, and has shown consistent results in multiple clinical populations [22-24]. A study investigating the impact of chemotherapy on cognitive functions of patients with BC demonstrated increased cognitive impairment throughout chemotherapy treatment, which did not recover 2 months after chemotherapy was completed [25]. In contrast, in the OGIPRO study, the cognitive performance of the patients receiving Ogivri showed potential improvement throughout the study treatment. However, due to the low number of cognitive tests recorded during app use, the cognitive abilities were analyzed descriptively and no association could be made with regard to the trastuzumab biosimilar treatment. Further analyses are needed to evaluate the electronically collected cognitive test results in patients treated with biosimilars and corresponding reference products.

Our study has several strengths and limitations. The limitations of the study included the design that lacked a prospective control group so that the study was not randomized. However, the comparison between prospectively collected data of patients treated with Ogivri and the historical ePRO data of patients treated with Herceptin in two previous studies [7,13] demonstrated no difference with regard to symptoms, well-being, and AEs. The earlier versions of the mobile app used in the historical cohort were developed to record symptoms and treatment side effects continuously and according to the CTCAE in patients with cancer, but were not designed to send questionnaires to patients. Nevertheless, the ePRO data of the historical cohort were recorded in the same way in the earlier versions of the mobile app [11] and are thus comparable to those of the prospective cohort. An exploratory analysis on cognitive abilities was performed only in the prospective cohort as these data were not available in the historical cohort. Further studies that are randomized and sufficiently powered to evaluate the real-word cognitive functions in patients with HER2-positive BC treated with anti-HER2 biosimilars are needed.

The major strength of our proof-of-concept study is that it was able to provide the first evidence that data collected via an autonomous eHealth app can also be used longitudinally to determine the similarity of a trastuzumab biosimilar to the reference product for the treatment of HER2-positive BC. Furthermore, our study has reached its primary endpoint, showing a similar average CTCAE score between patients treated with the trastuzumab biosimilar Ogivri and those treated with the reference drug Herceptin. Our results suggest that the use of a patient empowerment eHealth app in patients treated with anti-HER2 biosimilars is reliable and can support therapy management.

In conclusion, in patients with HER2-positive BC, treatments with the trastuzumab biosimilar Ogivri and the reference drug Herceptin resulted in equivalent symptoms, AEs, and well-being reported by ePRO. Hence, the integration of an ePRO tool into research and clinical practice can provide reliable information when investigating the real-world tolerability and safety outcomes of similar therapeutic compounds.

## Appendix

Multimedia Appendix 1 Multivariate analyses of Common Terminology Criteria for Adverse Events (CTCAE) scores (Table S1) and well-being scores (Table S2); distribution of cognitive performance scores (Figure S1).

## Abbreviations

AE: adverse event
BC: breast cancer
BW: body weight
CTCAE: Common Terminology Criteria for Adverse Events
ECOG PS: Eastern Cooperative Oncology Group Performance Status
ePRO: electronic patient-reported outcome
HER2: human epidermal growth factor 2
QoL: quality of life
SAP: statistical analysis plan
TMT: Trail Making Test

## Data Availability

The data sets generated and/or analyzed during this study are available from the corresponding author on reasonable request.
