# Supplementary material for: Comparison of the Real-World Reporting of Symptoms and Well-Being for the HER2-Directed Trastuzumab Biosimilar Ogivri With Registry Data for Herceptin in the Treatment of Breast Cancer: Prospective Observational Study (OGIPRO) of Electronic Patient-Reported Outcomes
Source: JMIR Cancer. 2024 Apr 4;10:e54178. doi: 10.2196/54178 (PMC11027054; doi:10.2196/54178)

# Multimedia Appendix 1

### Table S1: Linear Regression of CTCAE score – multivariate analysis

| **Parameter** | **Estimates** | **Standard Error** | ***P*-value** |
| --- | --- | --- | --- |
| **Prospective cohort vs historical cohort** | 2.51 | 2.951 | 0.398 |
| **Age (continuously)** | -0.37 | 0.127 | 0.004 |
| **T2 vs T1** | 14.99 | 4.11 | <0.001 |
| **T3 vs T1** | 10.8 | 4.7747 | 0.027 |
| **T4 vs T1** | 3.86 | 9.653 | 0.69 |
| **Neoadjuvant vs adjuvant setting** | -7.93 | 3.578 | 0.03 |
| **Palliative vs adjuvant setting** | 0.51 | 9.469 | 0.957 |

### Table S2: Linear Regression of well-being score – multivariate analysis

| **Parameter** | **Estimates** | **Standard Error** | ***P*-value** |
| --- | --- | --- | --- |
| **Prospective cohort vs historical cohort** | 3.78 | 4.293 | 0.382 |
| **Age (continuously)** | -0.2 | 0.188 | 0.29 |
| **T2 vs T1** | -12.66 | 6.057 | 0.04 |
| **T3 vs T1** | -15.24 | 7.027 | 0.033 |
| **T4 vs T1** | -17.48 | 14.261 | 0.224 |
| **Neoadjuvant vs adjuvant setting** | 18.57 | 5.217 | <0.0011 |
| **Palliative vs adjuvant setting** | 20.62 | 13.99 | 0.144 |

### Figure S1: Distribution of cognitive performances as execution time in seconds in the prospective cohort during study duration

Data of 37 out of 53 patients (70%) who performed at least one test were included in the analysis resulting in 767 cognitive tests entered in the app. Mean execution time was 42.9 seconds (SD 26.3; range 8-205 seconds).


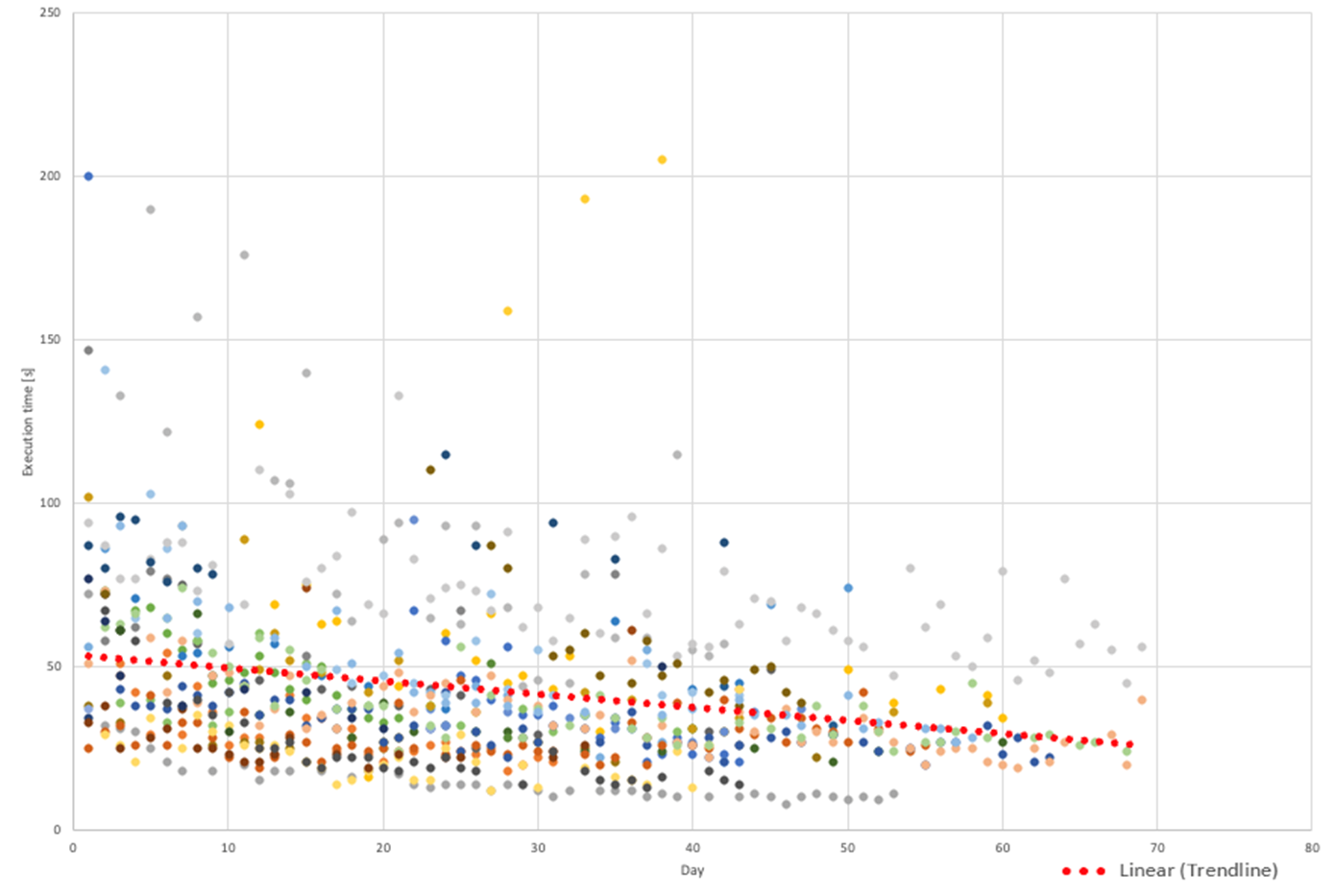

Supplement: Multimedia Appendix 1 [file cancer_v10i1e54178_app1.docx]
